# Quantitative Studies for Cell-Division Cycle Control

**DOI:** 10.3389/fphys.2019.01022

**Published:** 2019-08-19

**Authors:** Yukinobu Arata, Hiroaki Takagi

**Affiliations:** ^1^Cellular Informatics Laboratory, RIKEN, Saitama, Japan; ^2^Department of Physics, School of Medicine, Nara Medical University, Nara, Japan

**Keywords:** cyclin-dependent kinase, cyclin, transition probability, circadian oscillator, ultradian oscillator, metabolic oscillator, chaos, power law

## Abstract

The cell-division cycle (CDC) is driven by cyclin-dependent kinases (CDKs). Mathematical models based on molecular networks, as revealed by molecular and genetic studies, have reproduced the oscillatory behavior of CDK activity. Thus, one basic system for representing the CDC is a biochemical oscillator (CDK oscillator). However, genetically clonal cells divide with marked variability in their total duration of a single CDC round, exhibiting non-Gaussian statistical distributions. Therefore, the CDK oscillator model does not account for the statistical nature of cell-cycle control. Herein, we review quantitative studies of the statistical properties of the CDC. Over the past 70 years, studies have shown that the CDC is driven by a cluster of molecular oscillators. The CDK oscillator is coupled to transcriptional and mitochondrial metabolic oscillators, which cause deterministic chaotic dynamics for the CDC. Recent studies in animal embryos have raised the possibility that the dynamics of molecular oscillators underlying CDC control are affected by allometric volume scaling among the cellular compartments. Considering these studies, we discuss the idea that a cluster of molecular oscillators embedded in different cellular compartments coordinates cellular physiology and geometry for successful cell divisions.

## Introduction

Cells proliferate *via* rounds of cell division. Alma Howard and Stephen Pelc performed careful experiments, in which they labeled replicating chromosomal DNA in the meristem cells of fava beans (*Vicia faba*) using radioactive phosphorus-32 molecules. They deduced that each round of a cell cycle, including the Gap phase, DNA synthesis, and chromosomal segregation steps, has discrete phases (Howard and Pelc, 1951, 1953). This key study produced the concept of the *cell-division cycle* (CDC): namely, that cells divide after irreversible transitions through four discrete phases, G1, S, G2, and M (Pederson, 2003).

Cell biological and biochemical studies focusing on oocyte maturation and early embryonic cell division in various animal embryos (Masui and Markert, 1971; Wasserman and Masui, 1976; Evans et al., 1983; Hunt, 1989; Masui, 1992) and molecular genetic studies in budding and fission yeasts independently identified cell-cycle regulators (Hartwell et al., 1970; Nurse et al., 1976; Nurse and Bissett, 1981). Later, several independent studies using different model organisms together revealed an evolutionarily conserved core regulatory network for CDC regulation (Lee and Nurse, 1987; Arion et al., 1988; Hagan et al., 1988; Norbury and Nurse, 1989; Nurse, 1990). Together, these genetic and biochemical studies demonstrated a causal relationship between cell-cycle progression and underlying molecular reactions, eventually revealing that the network is formed by several feedback loops that regulate the kinase activity of cyclin-dependent kinases (CDKs) (Ferrell et al., 2011). These studies also revealed a network structure that recapitulated the oscillatory behaviors of CDK activity in mathematical models (Goldbeter, 1991; Norel and Agur, 1991; Tyson, 1991; Novak et al., 1998; Chen et al., 2000; Qu et al., 2003; Ferrell et al., 2011). Thus, a combination of qualitative and mathematical modeling studies has shown that the CDC is basically driven by biochemical oscillators centering on CDK kinases – in other words, by a CDK oscillator.

For successful cell division, multiple biochemical reactions must be spatiotemporally coordinated with intrinsic biochemical and biophysical conditions and extracellular environments by checkpoint control (Hartwell and Weinert, 1989; Weinert and Hartwell, 1989; Hartwell et al., 1994; Sherr, 1996; Paulovich et al., 1997). Checkpoint control is a mechanism for monitoring cell-cycle progression and ensuring the fidelity of genomic replication and spindle segregation. To ensure that there is time for the restoration and completion of earlier events, checkpoint control arrests or delays cell-cycle progression at the transition from G1 to S phase, G2 to M phase, or during S phase progression (Paulovich et al., 1997), a process known as the “arrest-or-go mechanism.” The total cell-cycle duration is determined by the basic period of biochemical oscillation and the time spent for checkpoint control.

On the other hand, quantitative observations have shown that the cell-cycle duration of genetically clonal cells exhibits reproducible non-Gaussian statistical properties. Although the basic oscillatory property of CDC control is supported by experimental and theoretical studies, the origin of the variability of CDC control remains unknown. Many successful studies of the mechanisms underlying the statistical property of time-evolving systems are found in the field of physics (Kuhn, 1962). These studies generally take the fundamental approach of measuring time-dependent changes in variables of interest, deriving the underlying statistical law, forming a hypothesis to explain this law, and experimentally verifying the hypothesis. Under the quantitative approach of seeking consistency between experimental results and models, a model is accepted after it is validated by experimental observations/perturbations and often after modification of the original model. This quantitative approach has also been effectively applied in the field of biology to understand system-level properties that cannot be reduced to the properties of individual molecular interactions.

This review examines quantitative CDC studies from a historical perspective, focusing on four topics: experimentally discovered statistical regularities in the (1) variability, (2) temporal correlation, and (3) frequency distribution of cell-cycle duration, as well as the (4) relation of cell-cycle duration with cell size. The review of these quantitative studies shows that the CDC is driven by a cluster of molecular oscillators, wherein the CDK oscillator is coupled with mitochondrial metabolic and transcriptional oscillators that operate in broad temporal frequencies spanning from minutes to a day.

Furthermore, this review discusses the discovery of, and models to account for, statistically reproducible distributions in CDC control. Statistical distributions in the CDC were often explained based on analogy to concepts in physics. Additionally, size-dependent CDC control in animal embryos exhibits scale-free dynamics. Recent studies have proposed that CDC dynamics are due to interactions of molecular oscillators embedded in geometrically different cellular compartments with a broad range of spatial scales, ranging from a micro-cluster in the mitochondrion to the nucleus or cytoplasm. We discuss how a cluster of molecular oscillators for CDC control functions to drive a cycle of chromosomal duplication and cell division in accordance with the cellular energetic/redox state and gene expression status. Interactions of molecular oscillators embedded in geometrically different cellular compartments may contribute to maintain a certain volume ratio among cellular compartments in cells of various sizes.

## Historical Development of Quantitative Studies for Cell-Division Cycle Control

### Variability and Statistical Regularity of Cell-Cycle Duration

During the 1960s, researchers developed several methods for synchronizing mammalian cell culture, including mechanical cell selection (Tobey et al., 1967), addition of chemicals to induce metabolic imbalance (Xeros, 1962), and serum deprivation (Burk, 1966). However, cell-cycle synchrony was difficult to maintain after one or more rounds of cell division (Petersen and Anderson, 1964; Burk, 1970; Minor and Smith, 1974). Long-term time-lapse recordings of CDC revealed that individual genetically clonal cells and siblings, which were expected to be biochemically and genetically identical, exhibited markedly different cell-cycle durations (Sisken and Kinoshita, 1961; Dawson et al., 1965). This heterogeneity was attributed to the complexity of sequential events. Specifically, researchers proposed that although sequential events are coordinated for successful division, there can be small differences between events in different siblings due to the complexity of the CDC process, and that the accumulation of these small differences can eventually lead to significant variability in cell-cycle duration (Minor and Smith, 1974).

**Figure 1:**
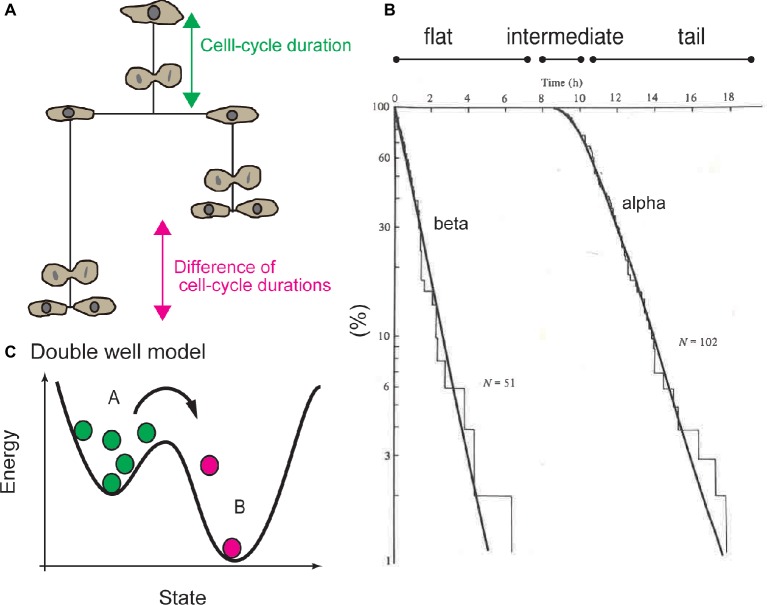
Exponential tail of distribution of cell-cycle duration and transition probability model. **(A)** Cell-cycle duration (the period from completion of the previous round of division or release from the quiescent state to the next round of cell division) or the difference between of cell-cycle durations between sister cells were used to study statistical property of CDC. **(B)** Cumulative histogram of cell-cycle duration, or waiting time distribution. Tail of the waiting time distribution is called the alpha curve. Distribution of the difference of the cell-cycle duration between sister cells is called the beta curve. Waiting time distribution is divided into the flat region, intermediate (or convex) region, and exponential tail. **(C)** Double well model to explain mechanism of transition probability model, the exponential tail of the waiting time distribution is interpreted based on the probability of reaction kinetics of each single molecule. For example, radioactive molecules (green circles) become nonradioactive (magenta circles) in a certain probability in a certain period. In this model, “old” radioactive molecules are equally likely to lose radioactivity as “young” molecules. Panel **(C)** is reused with permission from Cambridge University Press (Brooks, 1981).

On the other hand, Smith and Martin developed the transition probability model. Using this model, they proposed that the heterogeneity in cell-cycle duration can be attributed to a certain molecular mechanism in the CDC system (Smith and Martin, 1973, 1974). Generally, a cumulative histogram of the durations of the undivided state of cells or the cell-cycle duration will exhibit a distribution with a reproducible shape (Figures 1A,B). The waiting time distribution for cell division can be qualitatively classified into three regions: (1) the flat region, when no cell divides, (2) intermediate region, when some cells divide, and (3) tail region, when cells rapidly divide (Figure 1B). Smith and Martin proposed to fit the tail region with an exponential decay function, called the alpha curve (Figure 1B; Smith and Martin, 1973, 1974). The exponent of the alpha curve was the same as the exponent of the beta curve, which was determined by the difference of the cell-cycle duration between sister cells (Figure 1B; Shields and Smith, 1977; Shields et al., 1978). Exponents of the alpha and beta curves varied as a function of serum concentration (Shields and Smith, 1977). These experiments and the common values of the alpha and beta curves suggested that the CDC can be regulated by a mechanism associated with exponential behavior.

Exponential decay is observed in the primary kinetics of chemical reactions (Figure 1C). For example, the mass of radioactive molecules decreases in an exponential manner; *d*[*A*] / *dt* = −*k*[*A*] leads to [*A*] ∝ *e*^−*kt*^. Exponentiality is derived by stochasticity: the chemical reactions of each molecule occur in a certain probability at any time, independently of the history of the molecule. Due to the stochasticity in a single-molecule reaction (SSR), the waiting time until molecules lose their radioactivity exhibits an exponential distribution, which is explained by double well potential model (Figure 1C). In other words, an “old” radioactive molecule is equally likely to lose radioactivity as a “young” one, and one cannot determine which molecules will lose radioactivity in subsequent periods.

**Figure 2:**
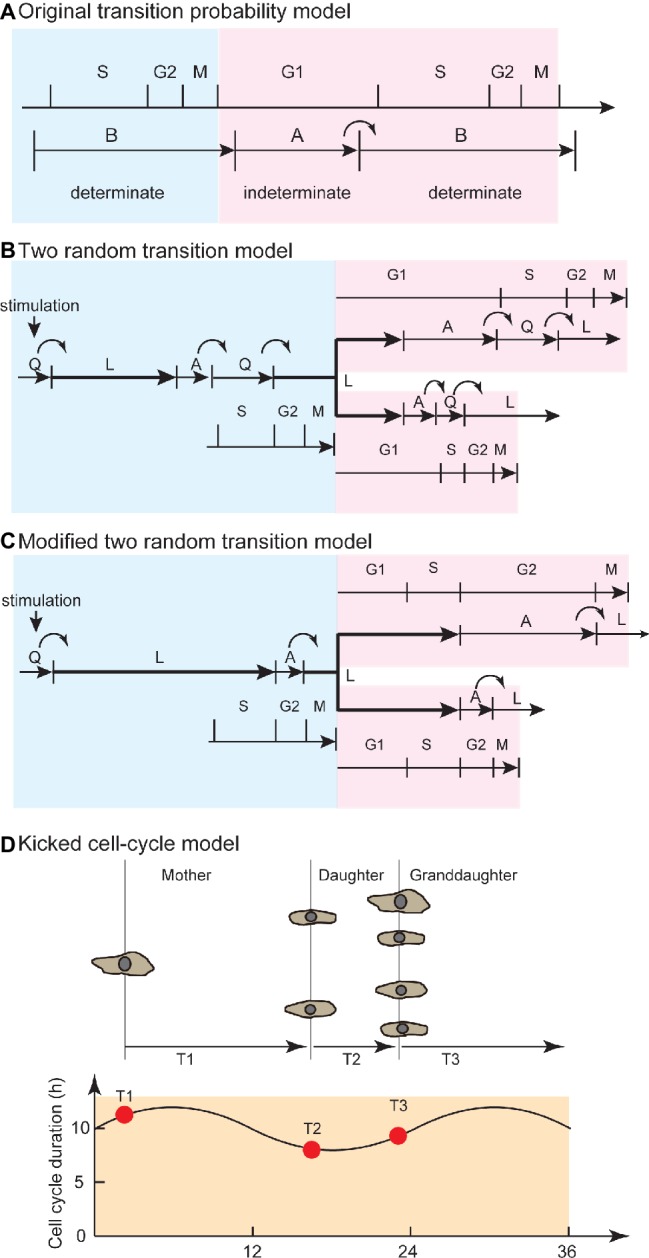
Evolution from the transition probability model to the kicked cell-cycle model to explain temporal correlation in cell pedigree. **(A)** Original transition probability model. The CDC is separated into A and B states, corresponding to the G1 and S/G2/M phases. The B state has a certain length of time, and transition from A to B occurs in a stochastic manner. This model explains the flat and exponential tail regions in waiting time distribution (alpha curve, Figure 1B). Blue area corresponds to mother cell, whereas red area corresponds to daughter cell. **(B)** Two random transition probability models. This model explains the exponential tail regions (alpha curve) in waiting time distribution and temporal correlation between mother and daughter cells due to QL and AQ stochastic transitions and overlapping L phase between mother and sister cells. Stochastic QL transition occurs at transition from serum-deprived quiescent state. Stochastic AQ transition occurs at the G1/S transition. Q-L-A sequence is maintained in quiescent and proliferating cells. **(C)** Modified two random transition models, with modifications to explain new experimental findings (van Wijk et al., 1977). Stochastic QL and AL transitions occur at G1 and G2 phase, respectively, to explain variability among unrelated cells and between sister pairs. L-A sequence is maintained in quiescent and proliferating cells. **(D)** Kicked cell-cycle model. To explain the high correlation of cell-cycle duration between mother and cousin cells, this model assumed an oscillator that has a longer period than the CDC. If the value of a component in the long oscillator at birth is high (or low), then the cells have a long (or short) cell-cycle duration. Due to the high value in mother (T1) and cousin (T3) cells at birth, the mother-cousin correlation is achieved. Panel **(A)** is modified from Smith and Martin (1973), whose copyright is in the public domain. Panel **(C)** is used by permission from Springer Customer Service Centre GmbH: Springer Nature, *Bulletin of Mathematical Biology*, Recent views on the cell cycle structure, (Bertuzzi and Gandolfi, 1983), with modification. Panel **(D)** is used by permission from Springer Customer Service Centre GmbH: Springer Nature, *Nature*, Lineage correlations of single cell division time as a probe of cell-cycle dynamics, (Sandler et al., 2015), with modification.

Smith and Martin explained the exponential property in waiting-time distribution of cell-cycle duration by analogy to SSR. To illustrate the transition probability model, the authors stated that “the transition from presumptive A phase to B phase in CDC is random, in a sense as if the radioactive decay is random” (Smith and Martin, 1973). Hence, the transition probability model implied that the timing of cell division is not determined by the time from the end of the previous round of division, or by the age of the cell, but by SSR. The model proposed that the CDC is qualitatively separated into presumptive A and B states. The B state has a constant duration, and the transition from A to B occurs in a stochastic manner, which corresponds to the flat and exponential tails of the waiting time distribution (Figure 2A). In this model, growth factors in serum regulate the probability of transition in cell-cycle progression (Shields and Smith, 1977).

Consistent with this model, experiments showed that cells cultured in various serum concentrations exhibited different slopes for the alpha curves (i.e., the probability of transition was changed by serum concentration) (Shields and Smith, 1977). Even in confluent culture, cells exhibited low transition probability, but the cell cycle was not arrested. Thus, the transition probability model supports probability regulation for cell-cycle progression, which does not support the “arrest” of cell-cycle progression at the quiescent G0 phase (Brooks and Riddle, 1988; Zetterberg et al., 1995; Ren and Rollins, 2004; Matson and Cook, 2017). The most striking aspect of the transition probability model is that the CDC is inherently a discontinuous process separated by SSR (Figure 1C).

Recently, it has been reported that variations in cell-cycle duration can be due to stochasticity caused by a low concentration of G1 cyclins (Di Talia et al., 2007). In a very low concentration solution, molecules rarely interact due to the small number of potentially reactive molecules. Rather than SSR, this stochasticity is caused by the stochasticity in a mass reaction (SMR). Under such low concentrations, the reaction time (e.g., until substrate decreases up to 1/*e* of the original concentration) varies for each experiment. The probability distribution of reaction times among experiments is distributed in a Gaussian rather than an exponential manner. When the CDC is driven by molecular regulators under SMR, the cell-cycle duration is also distributed in a Gaussian manner, which cannot explain the exponential distribution of waiting time for cell division. Therefore, the exponential tail causes a stochasticity that is different from the SMR. Although SMR is one factor that explains the variability of cell-cycle duration, it is distinct from the SSR hypothesized in the transition probability model.

To seek experimental evidence of stochastic transitions in the conventional CDC phases (G1, S, G2, and M), researchers studied the kinetics of cell-cycle phase transitions. Researchers probed for S-phase initiation by radioactively labeling mammalian cells in S phase using thymidine ([3H]-TdR) (Brooks, 1975) while visually observing bud formation of yeast cells, as a marker of S-phase initiation (Shilo et al., 1976). The number of cells in G1 phase (or S phase) decreased (or increased) exponentially. The A phase was attributed to most of the G1 phase, whereas the B phase represented the sum of the later cell-cycle phases (Figure 2A). Later, the transition probability model was expanded to explain the transition from serum-deprived quiescent Q state (QL transition) and the G1/S transition (AQ transition) by hypothesizing another random transition: namely, a CDC comprising two random transitions (Figure 2B; Brooks et al., 1980). However, further experimental observations using autoradiography and time-lapse recordings showed that exponential decay was observed not only in G1 but also in the G2/M transition (van Wijk et al., 1977). Observations of proliferating cells (Dowling et al., 2014) and of cultured cells that had been synchronized by thymidine deprivation (Ronning and Lindmo, 1981) showed that the G1 and S/G2/M phase durations were variable and exhibited exponential distributions. Thus, the exponential distribution was caused not only by G1/S transition but also by transitions at the other CDC phases.

A recent model incorporates this exponential property in multiple cell-cycle phases by using a delayed exponential function, with a constant duration before the start of the exponential tail in each phase, to express the durations of three cell-cycle phases (Weber et al., 2014). The total cell-cycle duration is determined by convoluting the three delayed exponential functions, which renders a delayed hypo-exponential function. The delayed hypo-exponential function provided a good fit to the previously reported waiting time distributions of slow- and fast-growing cell lines (Weber et al., 2014). Although it remains controversial whether the tail of the waiting time distribution can be fit with the exponential or hypo-exponential function, the delayed hypo-exponential model can be considered as the latest version of transition probability model.

Overall, the transition probability model is a pioneering quantitative approach in cell-cycle research that was developed by combining quantitative observations and mathematical model fitting to experimental data. However, this model has not generally been accepted as a standard model to characterize the cell-cycle duration. The biggest drawback of the model is that there are insufficient statistical tests to justify the exponential character of the tail from other models (Nelson and Green, 1981). Consequently, many alternative mechanisms have been proposed to explain the tail shape of the waiting time distribution (Castor, 1980; Koch, 1980; Wullstein et al., 1980; Murphy et al., 1984; Dowling et al., 2014). Additionally, recent molecular analyses have not been able to identify any molecular reaction that exhibits SSR. In an attempt to explain the molecular origin of SSR, duplication of the microtubule organizing center (MTOC) was proposed (Brooks et al., 1980) because the MTOC particle in a cell is present as a single pair. However, to our knowledge, no experimental evidence exists to support SSR in the kinetics of MTOC duplication.

Despite these drawbacks, the transition probability model addresses important issues about the molecular origin of the variable durations of cell biological events and the molecular and physiological states of the “quiescent state” as a counterhypothesis for the G0-arrest concept (Brooks and Riddle, 1988; Zetterberg et al., 1995; Ren and Rollins, 2004; Matson and Cook, 2017). To further discuss the origin of the variability of cell-cycle durations, we introduce other lines of quantitative research in the following sections and discuss their relation to the transition probability model.

### A Molecular Oscillator Slower Than the Cyclin-Dependent Kinase Oscillator

Strictly speaking, experimental findings of correlations of CDC duration in a pedigree do not fit with the assumptions of the transition probability model. Although Shields and Smith reported no correlation of cell-cycle durations between mother and daughter cells (Shields and Smith, 1977), their finding is not the general case. Other researchers observed positive correlations between sister pairs (Marin and Bender, 1966; Minor and Smith, 1974; CollyndHooghe et al., 1977; Murphy et al., 1984; Staudte et al., 1984; Klevecz, 1992) and both positive and negative correlations between mother-daughter pairs (CollyndHooghe et al., 1977; Staudte et al., 1984; Klevecz, 1992). Cell-cycle duration was also correlated in the third-degree cell pedigree (i.e., between mother-cousin pairs and between cousins) (Dawson et al., 1965; van Wijk and van de Poll, 1979; Staudte et al., 1984; Sandler et al., 2015). Interestingly, the correlations between mother and granddaughter cells were greater than them between mother-daughter pairs (referred to as mother-cousin correlation). The correlation in mother-daughter pairs can be explained by shared cytoplasmic constituents between mother and daughter cells, whereas the mother-cousin correlation suggests that another mechanism functions in a longer time scale than the single cell-cycle duration.

To explain the mother-daughter correlation, several authors proposed the existence of a cell-cycle phase overlapping mother and daughter cells (Figure 2B; Brooks et al., 1980; Bertuzzi and Gandolfi, 1983; Staudte et al., 1984). In the two random transition model described by Brooks, due to an overlapping and constant L phase, the mother-daughter correlation and the flat phase in the waiting time distribution (Figure 1B) were explained through extension of the transition probability model. An exponential distribution without a flat phase obtained from the difference between sisters (beta curve, Figure 1B) was explained by random transitions in daughter cells. Later, researchers reported experimental observations that the variation of cell-cycle duration among unrelated cells was due to the G1 phase, whereas the variation between sister pairs was due to the G2 phase (van Wijk et al., 1977). To account for this finding, the two random transition models were modified so that random transitions occurred in the G1 and late G2 phases (Figure 2C; Bertuzzi and Gandolfi, 1983). Throughout these models and their modifications, the idea of a phase overlapping between mother and daughters was employed to explain the temporal correlation of cell-cycle duration.

For the mother-cousin correlation (Dawson et al., 1965; van Wijk and van de Poll, 1979; Staudte et al., 1984; Sandler et al., 2015), van Wijk and van de Poll proposed a model in which the daughter cells inherited a “compensation factor” to account for the long cell-cycle duration of the mother cell, while the granddaughter cells exhibited the same state as the mother cell (van Wijk and van de Poll, 1979). More recently, the kicked cell-cycle model proposed that the CDC is affected by a presumptive oscillator that is independent of CDC and has a longer period than the cell-cycle duration (Figure 2D; Sandler et al., 2015; Mizrahi et al., 2016). This model successfully explained the compensation factor hypothesis between mother-daughter pairs as well as the mother-cousin correlation by hypothesizing a presumptive molecular oscillator with a period longer than the cell-cycle duration. In this model, the cell-cycle duration periodically becomes longer and shorter in a pedigree due to the oscillator. The circadian clock is used as the candidate mechanism for long-term oscillation (Mizrahi et al., 2016). For example, after hepatectomy, cell-cycle entry to restore liver mass is significantly affected by the time of the day when surgery is performed (Jaffe, 1954). This day-night effect of hepatectomy is impaired in circadian-defective *Cry* knockout mice (Matsuo et al., 2003). Quantitative analysis based on continuous observation of cell cycle and clock gene expression showed that the circadian clock gates the timing of cell division (Nagoshi et al., 2004). Thus, the CDC is regulated by coupling with the circadian clock, an oscillator that is slower than the CDC.

As discussed above, mother-cousin correlation was explained by coupling of CDK oscillator with a slower oscillator than CDC like circadian oscillator. The other patterns of temporal correlations can be explained by this coupling model. When cell cycle duration is compatible to the period of circadian rhythm, cell cycle durations of mother and daughter cells can be correlated positively. When cell cycle durations are so long that stochastic variation of the cell cycle durations is significantly larger than the period of circadian rhythm, cell cycle durations of mother and daughter cells can be uncorrelated. Thus, coupling of CDK oscillator with circadian oscillator has a broader spectrum to explain temporal correlation in pedigree.

### A Molecular Oscillator Faster Than the Cyclin-Dependent Kinase Oscillator

**Figure 3:**
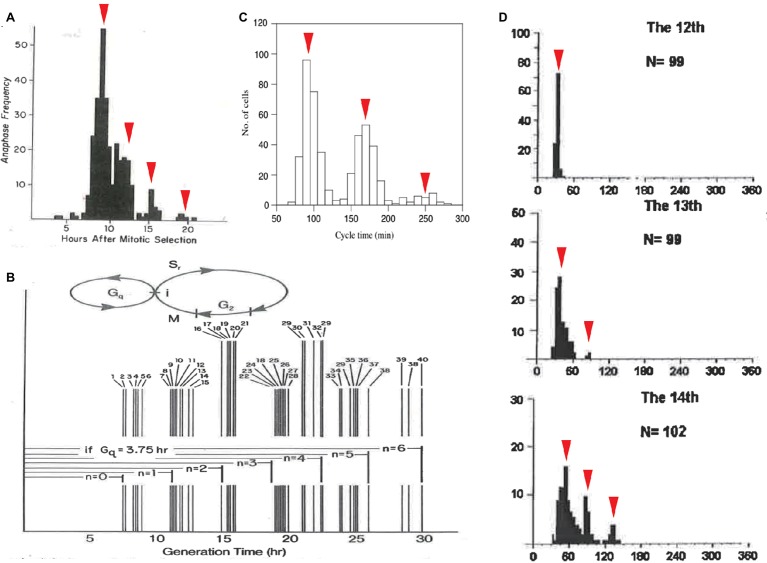
Quantized control of the CDC in various organisms. Quantized cell-cycle duration in mammalian cell lines **(A,B)**, in fission yeast mutant cells **(C)**, and in *Xenopus* embryonic cells **(D)**. Cell-cycle duration was elongated at an interval of 3–4 h in mammalian cells **(A,B)** and 60–70 min in fission yeast cells **(C)**. Cell-cycle duration at 13th, 14th, and 15th cleavage stages after the first cell cleavage was elongated at an interval of 30 min in *Xenopus* embryos **(D)**. Panels **(A,B)** are reused from (Klevecz, 1976), whose copyright is in the public domain. Panel **(C)** is reproduced with permission from Figure 6 in Sveiczer et al. (1996). Copyright permission of Panel **(A)** (Wang et al., 2000) was obtained from John Wiley & Sons, Inc. Arrowheads are added as a modification.

Cell-cycle duration exhibits another statistical regularity: quantization. Based on a manually obtained frequency histogram (Klevecz, 1976), Robert Klevecz discovered that the cell-cycle durations of 304 synchronized cells were distributed in a multimodal peak within a certain period of time. Specifically, the histogram was a discrete series of subpopulations interspersed by the intervals of 3.5–4 h (Figure 3A). Noting the constant interval, Klevecz described the cell-cycle duration as “quantized.” This concept is borrowed from the field of quantum physics, wherein photons and electrons have discrete (quantized) energy states. Klevecz demonstrated quantization in the cell-cycle durations of various mammalian cell lines (Figure 3B). This characteristic was later confirmed by Fourier analysis of histograms obtained by automated digital image analysis (Eccles and Klevecz, 1986). The distribution has also been reported in a fission yeast mutant, in *Paramecium tetraurelia* (Figure 3C; Kippert, 1996; Sveiczer et al., 1996) and in *Xenopus* embryo cells (Figure 3D; Masui and Wang, 1998; Wang et al., 2000). Thus, quantization of cell-cycle duration is a conserved property of eukaryotic CDC.

To explain the underlying mechanism of quantization, Klevecz proposed that there is a cellular process that cycles with a period shorter than CDC. This short oscillator is only allowed to gate timing to enter CDC (Figure 3B). Phase response curves obtained from several experimental perturbations supported the presence of another oscillator with a period consistent with the time interval between subpopulations (Klevecz et al., 1980; Shymko and Klevecz, 1981). An important question concerns the molecular identity of the faster oscillator. Such biochemical oscillations with periods shorter than a day, *c.f.,* circadian, are called ultradian (Edmunds and Adams, 1981; Kippert and Lloyd, 1995; Richard, 2003).

Long-term continuous culture of budding yeast was originally established for alcohol fermentation and later developed for academic research (Finn and Wilson, 1954; Maxon, 1954). In long-term aerobic continuous culture, medium is supplied and excess medium is withdrawn from a stirred tank, with yeast cells culturing at a constant rate, thereby maintaining the culture volume. During culture, autonomous ultradian respiratory oscillations of oxygen and carbon dioxide spontaneously emerge (Finn and Wilson, 1954; Kaspar von Meyenburg, 1969; Mochan and Pye, 1973; Richard, 2003). Concentrations of NAD(P)H, ATP:ADP, H_2_S, and the main cellular redox buffer, glutathione, oscillate with periods spanning from a few minutes to several hours (Satroutdinov et al., 1992; Murray et al., 1998, 1999, 2007; Roussel and Lloyd, 2007; Sasidharan et al., 2012). The amount of cellular NAD(P)H increases in the oxidative phase when mitochondria are more active (Sasidharan et al., 2012), whereas cellular H_2_S, a potent inhibitor of the respiratory chain *via* reversible binding with mitochondrial cytochrome c oxidase, increases when mitochondria are more inactive (Sohn et al., 2000). Oscillations are characterized by two phases, one of high oxygen consumption and one of low oxygen consumption, where oxidative and reductive processes, respectively, resulting from redox-energetic processes occur (Tu et al., 2005; Slavov et al., 2012; Mellor, 2016). Researchers observed metabolic oscillations, with periods of 3–4 h, in the activities of lactate hydrogenase and glucose-6-phosphate dehydrogenase in mammalian cells (Klevecz and Ruddle, 1968; Klevecz, 1969). More recently, an ultradian metabolic rhythm was described in mouse liver (Zhu et al., 2017). Indeed, examples of ultradian metabolic oscillations can be found throughout the literature in all branches of life (Lloyd and Murray, 2007; Brodsky, 2014; Mellor, 2016). Thus, ultradian metabolic oscillations appear to be evolutionarily conserved and, as such, are possible candidates for the faster oscillator hypothesized by Klevecz (1976).

Intriguingly, the CDC of budding yeast under conditions of aerobic continuous culture is autonomously synchronized without experimental manipulation (Kaspar von Meyenburg, 1969; Klevecz et al., 2004). Addition of glutathione (Murray et al., 1998, 1999), NaNO_2_ (Murray et al., 1998), acetaldehyde (Richard et al., 1996), or H_2_S (Sohn et al., 2000) perturbs the synchronization of metabolite oscillation. Thus, this synchronization is mediated by metabolites that are diffused in the culture media. CDC events, such as entry into S phase or cell division, occur in specific respiratory states (Kaspar von Meyenburg, 1969; Munch et al., 1992; Klevecz et al., 2004). Using the latest techniques for measuring single-cell concentrations of NAD(P)H and ATP, Papagiannakis et al. showed that the CDC is regulated *via* coupling of metabolic oscillators (Papagiannakis et al., 2017). To demonstrate the coupling, authors tested whether cellular and biochemical responses to various experimental perturbations can be explained from the general properties of a coupled oscillator predicted by a mathematical model. Coupling occurred at two distinct phases, G1 and S, regulating CDC. Thus, one of the candidate molecular systems for the faster oscillator hypothesized by Klevecz (1976) is a metabolic oscillator.

### A Scale-Free Mechanism to Cell Size for Cell-Division Cycle Control in Animal Embryos

Cell divisions occur in a balance with cell growth. Theoretically, according to mass action theory, chemical reactions in a well-mixed solution are independent of the solution size. Therefore, how cells measure their own size and balance their growth and division *via* biochemical reactions remain important questions. The titration model was proposed to explain the molecular mechanism of the cell size-dependent control of CDC (Amodeo and Skotheim, 2016). Titration is a technique for quantifying the number of molecules by using the known concentration of a different molecular species *via* a chemical reaction. For example, in acid-base titration, the concentration of an acid or base is determined by neutralizing a standard acidic or basic solution. In the titration model for cell size-dependent CDC control, the intracellular amount of a sensor molecule for cell size is measured by the amount of a protein as the standard (Schmoller et al., 2015; Amodeo and Skotheim, 2016).

In budding yeast, the amount of the G1 cyclin Cln3 increases in proportion to the cell size, whereas the amount of the cell-cycle inhibitor Whi5 is constant during G1. When the amount of Cln3 exceeds the amount of Whi5, transcriptional repression *via* Whi5 for S-phase initiation is released, and eventually CDC starts. Thus, the critical cell-size threshold for S-phase initiation is monitored by titration of Cln3 by Whi5 (Schmoller et al., 2015; Amodeo and Skotheim, 2016). In *Xenopus* embryos, the timing of mid-blastula transition (MBT), which initiates cell-cycle elongation by incorporating the Gap phase in the embryonic S/M cycle in CDC, and somatic transcription (Newport and Kirschner, 1982a,b) are cell size-dependent processes that are explained by the titration model (Collart et al., 2013; Amodeo and Skotheim, 2016). The titration model has also been applied to spatially localized size sensors in fission yeast (Martin and Berthelot-Grosjean, 2009; Moseley et al., 2009; Amodeo and Skotheim, 2016). Thus, the titration model is a widely applicable principle for cell size-dependent CDC control.

**Figure 4:**
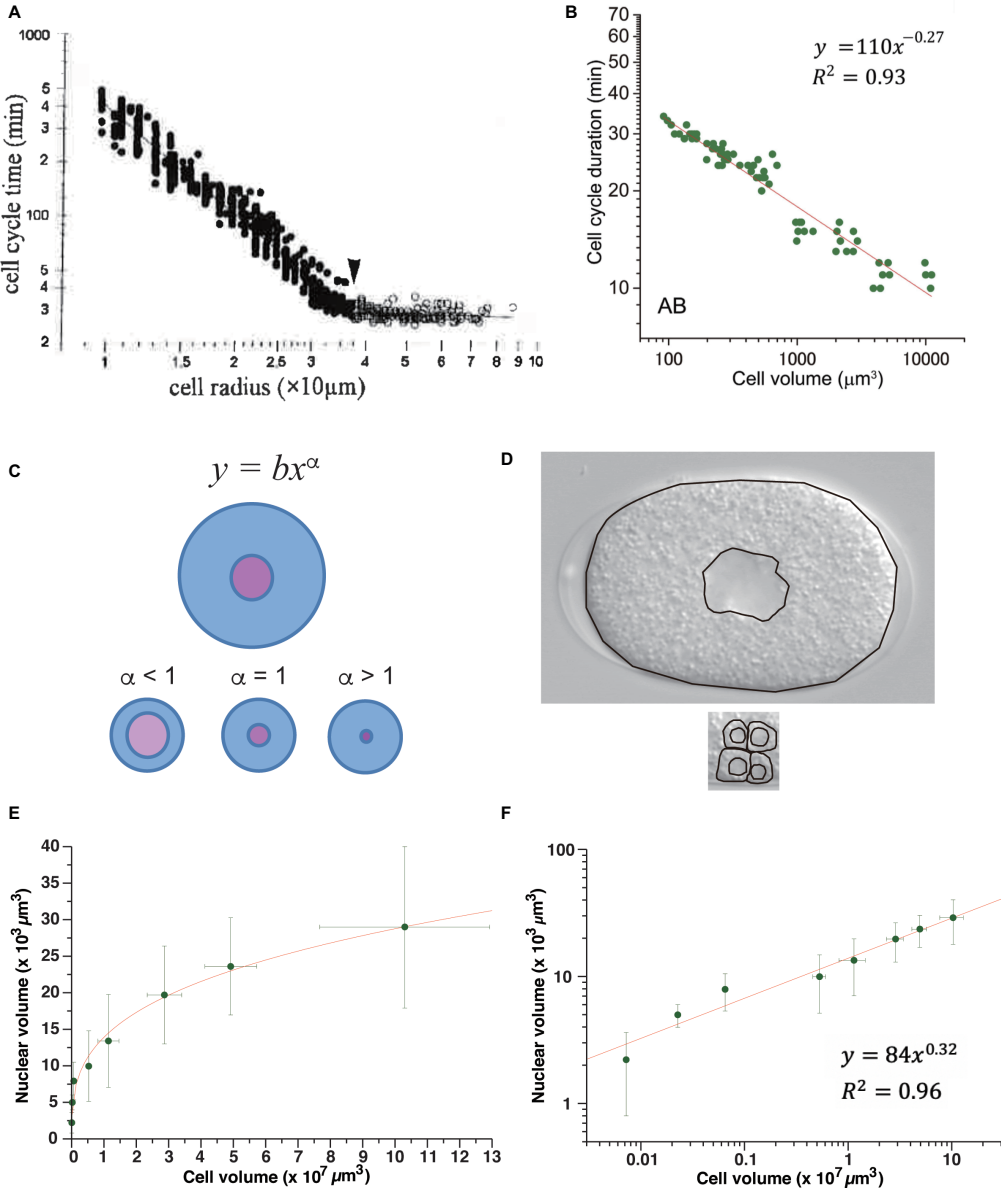
Power-law relationship between cell-cycle duration and cell size in animal embryos. Power-law distribution between cell-cycle duration and cell size in *Xenopus* embryos **(A)** and *C. elegans* **(B)** embryos. **(C)** Allometric scaling between nucleus and cell volumes in *C. elegans* embryos. One-cell embryo (upper) and cells at later embryonic stage (lower). Scaling exponent of 0.63 in *C. elegans* volume relation between nucleus and cell (Arata et al., 2014) was consistent with schematic illustration of alpha <1 (see panel **D**). **(D)** Images obtained from one-cell embryo (upper) and part of a ~100-cell embryo (lower) by differential interference contrast microscope. Boundaries of cell and nucleus are traced by black lines. Compare ratio of nuclear and cell areas between one-cell embryo and part of ~100-cell embryo. **(E,F)** Allometric scaling between nuclear and cell volumes after cell cleavages during *Xenopus* early development. Average values and standard deviation at each stage were extracted from graph plot in Figure 1B in (Jevtić and Levy, 2015). Data are shown in a linear plot **(E)** and log-log plot **(F)**. Fitting was performed by linear least-squares method in log-log graph. Copyright permission of Panel **(A)** (Wang et al., 2000) was obtained from John Wiley & Sons, Inc. Panel **(B)** is reused due to Creative Commons license CC-BY (Arata et al., 2014).

After MBT in *Xenopus* embryos, the duration of the cell cycle extends in correlation with the reduction in cell size due to cell cleavage. Wang and Masui studied the relation between cell-cycle duration and cell size in *Xenopus* embryonic cells isolated and cultured *in vitro* (Masui and Wang, 1998; Wang et al., 2000). They measured cell radius because the vertical and lateral views of isolated cells were round, indicating that the cells were almost perfectly spherical. Cell-cycle duration exhibited a power-law relation to the cell radius after MBT, with a power-law exponent of 2 (Figure 4A). This result suggested that *Xenopus* embryonic cells do not have any specific size that triggers cell division after MBT, or they have a scale-free mechanism to maintain cell size for cell-cycle control. Because the exponent was 2, the authors proposed a model in which mitosis promoting factor (MPF), which is mainly composed of cyclin B/Cdk1 (Masui, 1992), was produced in proportion to the cell surface area, and mitosis was initiated when the amount of MPF exceeded the amount of inhibitor present in proportion to genome size at the one-cell embryo stage (Masui and Wang, 1998; Wang et al., 2000). They proposed that “MPF is neutralized when it titrates a nuclear inhibitor.” Therefore, the *Xenopus* cell-surface hypothesis can essentially be classified as a titration model.

To test whether the cell surface hypothesis can be applied in other animal embryos, we studied the *Caenorhabditis* *elegans* time-volume relation, that is, the relation between the cell-cycle duration and cell volume of normally developing embryonic cells in an invertebrate, *C. elegans* (Arata et al., 2014). Cell volume was measured by making a finite integration of the cell area, observed by changing the focus of the microscope at a constant interval. Cell-cycle duration exhibited a power-law relation with cell volume. The absolute power of the *C. elegans* time-size relation varied depending on cell lineage and was classified into at least into three groups (−0.41, −0.27, and <0.13 in volume, and −1.2, −0.81, and <0.39 in radius). None of the exponents coincided with the exponents in *Xenopus* (Figure 4B); thus, the *C. elegans* time-size power-law relationship cannot be explained by the cell surface hypothesis.

The changeable nature of the time-size power-law exponents among cell lineages in *C. elegans* and the difference between *C. elegans* and *Xenopus* suggest that a flexible mechanism (rather than a simple physical constraint of cell geometry) may underlie the scale-free property of CDC control in animal embryos. Based on our experimental finding of allometric scaling between cell size and nuclear size in *C. elegans* (Arata et al., 2014), we considered the possibility that cell-cycle duration is determined by an interaction between biochemical reactions in different cellular compartments whose volumes are allometrically scaled. Our experiments showed that the relation between the nucleus and cell volume scaled allometrically, with a power-law exponent of 0.63 (see Figures 4C,D for an intuitive understanding of power <1). Allometric scaling between nuclear size and cell size has also been observed in *Xenopus* embryos (see discussion below; Figures 4E,F; Jevtić and Levy, 2015). By contrast, in budding yeast (correlation coefficient *r* = 0.65–0.82) (Jorgensen et al., 2007) and fission yeast (*r* = 0.97) (Neumann and Nurse, 2007), nuclear size scaled linearly with the cell size in the population average; therefore, the ratio was constant. Thus, allometric scaling between nuclear size and cell size is only shared in animal embryonic cells.

Interestingly, we found that the power-law exponent in the volume ratio between the nucleus and cell (−0.37; $\frac{V_{n}}{V_{c}}=\frac{{V_{c}}^{0.63}}{V_{c}}={V_{c}}^{-0.37}$) approximately coincided with the power-law exponent of the *C. elegans* time-size relation in the high-power group (−0.41; $T\propto {V_{c}}^{-0.41}$). This result is consistent with a model that *C. elegans* cell-cycle duration is determined in proportion to the volume ratio between nucleus and cell. We proposed that this match in power-law exponents in *C. elegans* embryos could be explained by a small modification of the titration model proposed by Wang and Masui. Assume that *C. elegans* MPF is produced in proportion to cytoplasmic volume (instead of the cell surface-proportional synthesis of MPF proposed in *Xenopus*), and that the nuclear MPF is the functional concentration. The nuclear concentration of MPF changes allometrically due to allometric volume scaling between the nucleus and cell. Thus, the scale-free nature of CDC control in *C. elegans* can be explained by a combination of the cell volume-dependent synthesis of MPF and allometric volume scaling between the cell and nucleus, where MPF is produced and functions, respectively. We refer to this model as the allometric titration model.

As nuclear size is genetically controlled (Levy and Heald, 2010), the allometric titration model appears to be more applicable to explain the changeable nature among cell lineage in *C. elegans* and between *C. elegans* and *Xenopus* embryos. In addition, cell volume-dependent production of cyclin is consistent with that of Cln3 in budding yeast (Schmoller et al., 2015). Conventionally, the nuclear-cytoplasmic ratio or karyoplasmic ratio has been considered to be a measure of cellular spatial information (Wilson, 1925; Jorgensen and Tyers, 2004). Previous researches proposed that the nuclear-cytoplasmic ratio dictates cell size-dependent CDC control in budding yeast and MBT triggering in *Xenopus* embryos (Newport and Kirschner, 1982a; Futcher, 1996) and altered size ratio can affect cell and organismal growth and development, and is associated with disease states (Edens et al., 2013; Jevtić and Levy, 2015; Jevtić et al., 2019). However, quantitative analyses of the allometric size scaling between cell and nucleus have been limited to studies using *C. elegans* and *Xenopus* embryos in animal cells (Arata et al., 2014; Jevtić and Levy, 2015). The allometric titration model incorporates previous models for various organisms and provides a more quantitative view for the original titration model.

Allometric volume scaling of the nucleus and cell provides a new clue to explain the different power-law exponents of *Xenopus* and *C. elegans* embryos. It is worth testing whether this model can explain the scale-free CDC control in *Xenopus* embryos. If the allometric volume scaling model has a generality, then we can predict the scaling exponent *α* between nucleus volume and cell volume after MBT in *Xenopus* should be 0.33 ($\frac{V_{n}}{V_{c}}=\frac{{V_{c}}^{\alpha }}{V_{c}}=r^{-2}={V_{c}}^{-2/3}$, then *α* = 0.33). If this prediction is valid, then the presumptive experimental data are consistent with a model that scale-free CDC control in *Xenopus* embryos is caused by the volume-dependent synthesis of MPF and allometric volume scaling between nucleus and cell. However, the predicted scaling exponent 0.33 indicates that the nuclear size is hardly changed by a reduction in cell size; the rate of change in the reduction of nuclear size to cell size is much lower than for *C. elegans*. To know if our hypothesis deserves to be tested, we studied the volume relationship between the nucleus and cell in *Xenopus* using published data (Jevtić and Levy, 2015). We found that the nuclear volume and cell volume *before* MBT in *Xenopus* were allometrically scaled, and the power exponent was 0.32 (Figures 4E,F), indicating that such a low exponent was possible in *Xenopus* embryos. More importantly, the exponent of 0.32 *before* MBT approximately coincided with our predicted scaling exponent of 0.33. Therefore, the allometric titration model may deserve to be tested in *Xenopus* embryos *after* MBT.

## Building a Unified Model of the Quantitative Studies for Cell-Division Cycle Control

As discussed, four lines of historical developments of quantitative CDC studies, namely the (1) variability of cell-cycle duration, (2) temporal correlation of cell-cycle duration, (3) quantization of CDC control, and (4) power relation between cell size and cell-cycle duration, have been independently developed. For each topic, a model has been developed that sufficiently explains the individual statistical properties. In this section, to accommodate a broader range of experimental observations, we discuss how these models are related and seek a unified view of CDC control. Multiple experimental and theoretical studies showed that CDK oscillator is coupled with circadian, metabolic, and transcriptional oscillators (introduced below) to form a clustered oscillator and generates chaotic dynamics for CDC control. We also discussed how the chaotic oscillator is regulated by intracellular geometry to drive CDC. Finally, we discussed the evolutional origin of the clustered oscillator.

### A Deterministic Chaotic Model

An important question raised by the transition probability model is whether the variable cell-cycle duration originates from a stochastic or a deterministic mechanism. Even a simple multistep sequential biochemical reaction reproduces an exponential distribution (Bel et al., 2009), which implies that the exponential tail of the waiting time distribution is not necessarily caused by SSR. Oscillator models have also been used to explain the exponential tail of waiting time distribution (Shymko and Klevecz, 1981; Gilbert, 1982; Klevecz and Shymko, 1985). In models where a fast and slow oscillator is coupled, it is presumed that cells on the fast oscillator transit to the slow oscillator by “noise” and thereafter divide (Shymko and Klevecz, 1981). When multiple oscillators are tandemly integrated before the transition to the slow oscillator, the number of the undivided cells gradually decays, then the model recapitulates the exponential decay of undivided cells by “a quasi-exponential tail” (Klevecz and Shymko, 1985).

**Figure 5:**
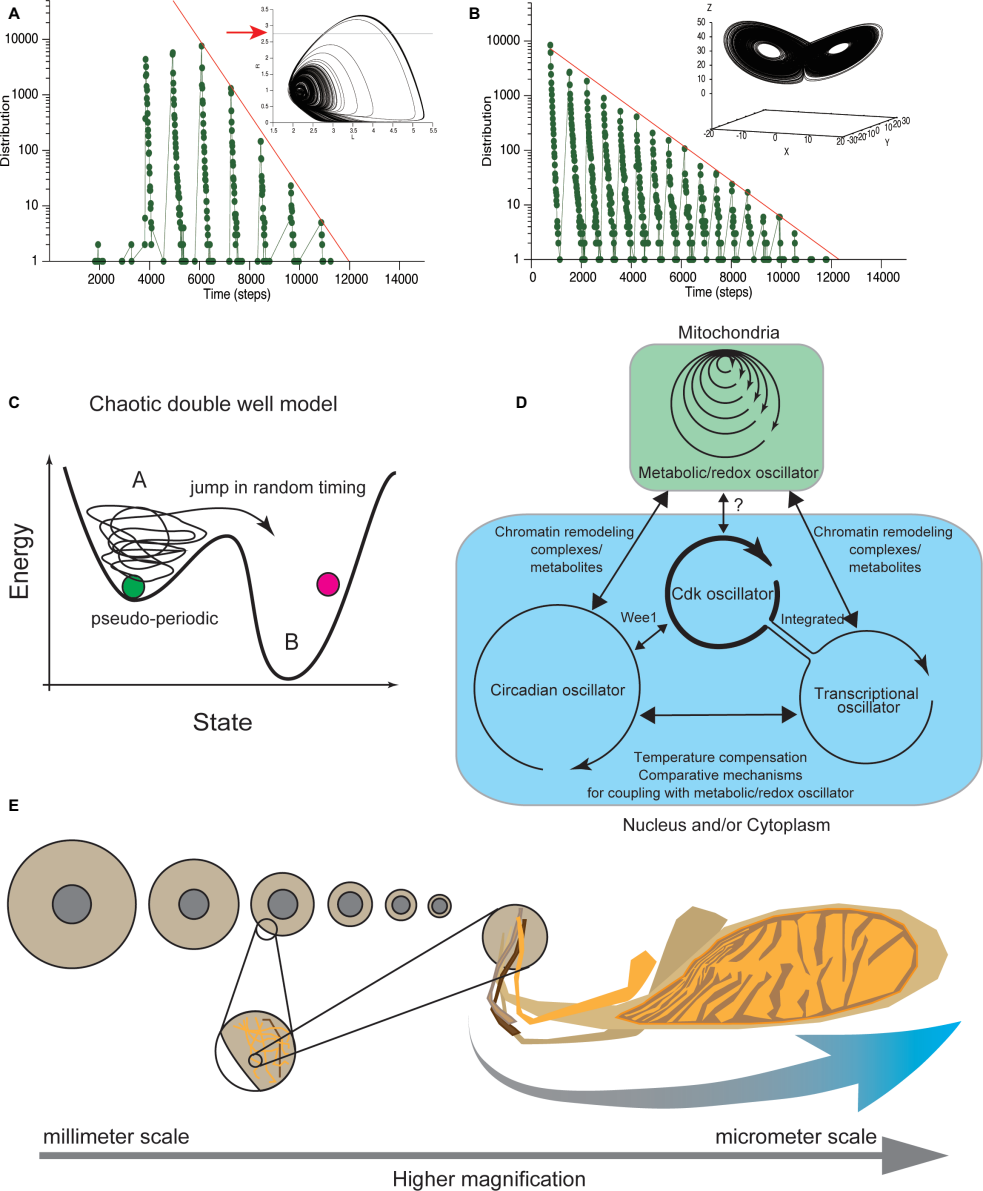
Clustered oscillator for CDC control embedded in different cellular compartments. **(A)** Cell-cycle durations calculated from Lloyd model (Lloyd et al., 1992) distributed in locally periodic and globally exponential manners. Temporal changes of two variables shown in two-dimensional (phase) space. Coordinate defined by variables follows a characteristic pseudo-periodic orbit (upper), assumed to represent the cellular physiological state. Residence times, when the coordinate is below the threshold (red arrow in upper panel), exhibits an exponential distribution, confirmed in our simulation (lower). **(B)** In the Lorenz model, temporal changes of three variables are shown in three-dimensional phase space. Coordinate defined by the variables follows a characteristic orbit with two pseudo-periodic cycles (upper). Residence times in one side of the cyclic orbits distributed in a locally periodic and globally exponential manner (lower). Exponential distribution of residence times was confirmed in our simulation under the previously reported parameter set (Anishchenko et al., 2004). **(C)** Chaotic transition in double-well potential. Theoretical deterministic chaotic models **(A,B)** reproduce both periodic and exponential distributions of cell-cycle duration. These chaotic dynamical models explain that the physiological state of cells changes pseudo-periodically, and cells eventually divide after the physiological state exceeds a threshold **(A)**. Transition to the other region occurs with a deterministic but random chaotic nature. In this new double well model, biochemical systems that control at least G1 and G2 (van Wijk et al., 1977) have chaotic dynamics. **(D)** CDK oscillator is coupled with metabolic, transcriptional, and circadian oscillators to drive the CDC. Metabolic oscillator has a multiple time scale period that functions in mitochondria (blue). The other oscillators function in nucleus and/or cytoplasm (green). The relation among metabolic, transcriptional, and circadian oscillators (black arrows) with the CDK oscillator is discussed in the main text. The CDK oscillator may coordinate cell-cycle progression based on information about cellular physiological state received from the metabolic and transcriptional oscillators. **(E)** Broad spectrum of size where the biochemical clustered oscillator for CDC control functions. Allometric scaling between nuclear size and cell size. In *C. elegans* embryos, the relative size of nucleus to cell size allometrically scaled (left). Mitochondrial cluster has a scale-free structure (right). Coupling of biochemical reactions that occurs in different organelles can be source of information about the relative sizes of cellular organelles to the cell size, or cellular geometry.

Lloyd and colleagues later showed that when coupling an ultradian oscillator and a slower oscillator, the model shows chaotic dynamics (Lloyd et al., 1992). The deterministic chaotic dynamical system enhances a minute difference in the initial variables exponentially. Eventually, the temporal change of variables becomes unpredictable or appears random. Thus, the “noise” assumed in the previous model was defined by the randomness inherent in a chaotic dynamical system. By contrast, in the Lloyd model, the cell-cycle duration (the time when a set of variables in the model resides in a region within a certain threshold) becomes random depending on the chaotic nature (Figure 5A). This variability of cell-cycle duration in the model recapitulates the variation around each of quantized cell-cycle durations observed in the experiments.

We performed a simulation under these conditions (Lloyd et al., 1992) and confirmed that the cell-cycle durations globally exhibited an exponential distribution (Figure 5A). Therefore, the Lloyd model explains both the quantization property and the exponential tail distribution of cell-cycle duration. In addition, two representative chaotic dynamical models, the Lorenz model and Duffing model, show that a set of variables changes in an orbit with two different cyclic regions (Figure 5B; Bergé et al., 1986). Researchers have studied the residence time of variables on one side of the cyclic regions and showed that the residence time distribution exhibited both local periodic and globally exponential properties (Nicolis et al., 1993; Anishchenko et al., 2004). We also confirmed the global exponential distribution in our simulation under the previously reported parameter set (Figure 5B; Anishchenko et al., 2004). Each of the periodic and exponential natures reproduced in these deterministic chaotic models, including the Lloyd model, derive from the balance of the period of a single oscillator and the time scale of the random nature of chaos, which is explained by chaotic double well potential model (Figure 5C). Thus, the exponential and periodic natures reproduced in these models are found in many chaotic dynamical systems and are generic properties of deterministic chaotic models.

Recently, Sandler et al. tested whether the CDC is controlled by a stochastic or deterministic mechanism, by combining experimental measurements with nonlinear data analysis (Sandler et al., 2015). They measured the cell-cycle duration in thousands of cells along several generations. Their findings supported the deterministic control of CDC. Taken together, these findings show that CDC is controlled by a deterministic chaotic dynamical system, rather than by a stochastic mechanism defined by SSR. This deterministic chaotic model of CDC control provides a consistent picture obtained from a wide range of experimental observations focusing on variability, temporal correlation, and quantized control of CDC (Figure 5D).

### Molecular Mechanisms to Couple Multiple Oscillators and Their Evolutionary Relationships

The metabolic oscillator and mammalian circadian oscillator are coupled with the CDK oscillator and involved in CDC control. In this section, we introduce another oscillator that is coupled with the CDK oscillator for CDC control. We also discuss the molecular mechanisms for oscillation and coupling of these oscillators, as well as the evolution of this cluster of molecular oscillators for CDC control.

#### Molecular Mechanisms for Coupling Three Molecular Oscillators With the Cyclin-Dependent Kinase Oscillator

Although both the budding yeast metabolic oscillator and mammalian circadian oscillator are not required for CDC, they do gate the cell-cycle period (Nagoshi et al., 2004; Papagiannakis et al., 2017). The mammalian circadian oscillator is coupled with the CDK oscillator through circadian clock-regulated expression of a conserved cell-cycle regulator, the Ser/Thr kinase Wee1 (Figure 5D; Matsuo et al., 2003; Reddy et al., 2005). On the other hand, the direct mechanism to couple a metabolic oscillator with the CDK oscillator at the G1 and S phases remains unclear (Figure 5D; Papagiannakis et al., 2017). Interestingly, the oscillatory dynamics of NAD(P)H and H_2_S in a continuous culture of budding yeast has a broad temporal range, from several minutes to hours (Figure 5D; Roussel and Lloyd, 2007; Sasidharan et al., 2012), with a power spectrum that exhibits a power-law distribution (Sasidharan et al., 2012).

Results from nonlinear time series analysis suggested that the scale-free nature of metabolic dynamics is caused by the chaotic solution of a deterministic biochemical reaction system (Roussel and Lloyd, 2007; Aon et al., 2008), which may consist of the interaction of the biochemical system and fractal structure of mitochondria (Aon et al., 2004a, 2006; Kurz et al., 2010, 2014). Mitochondrial cluster size experimentally showed a power-law or fractal spatial distribution (Aon et al., 2004b). The oscillatory frequency in the inner membrane potential of mitochondria in cardiac myocytes was correlated with the size of mitochondrial clusters and showed a power-law relationship with cluster size (Kurz et al., 2015). To explain these experimental observations, it was proposed that an oscillatory reaction in a large mitochondrial cluster takes longer to synchronize metabolic reactions within the micro-cluster compared to a reaction in a small cluster, due to the longer diffusion time (Kurz et al., 2010, 2015). Thus, scale-free temporal dynamics of mitochondrial metabolic reactions are thought to be caused by the self-similar and scale-free nature of the size of the reaction space in mitochondria. Metabolic dynamics are under the geometric control of the cellular organelle. How the scale-free mitochondrial metabolic dynamics interact with the CDK oscillator and other oscillators is an interesting issue (Figures 5D,E).

In addition to metabolic and circadian oscillators, experimental studies using budding and fission yeast have suggested the presence of another molecular oscillator critical for CDC control: a transcriptional oscillator (Novak and Mitchison, 1986, 1987, 1990; Novak et al., 1988; Haase and Reed, 1999). Cell cycle-arrested budding yeast lacking multiple mitotic and S-phase cyclins continued to show periodic activation of subsequent G1-specific events that were essential for cell-cycle progression (Haase and Reed, 1999). Comprehensive microarray studies of budding yeast in aerobic continuous culture revealed that more than half of genes in the budding yeast genome are expressed periodically, in accordance with respiratory oscillations (Spellman et al., 1998; Klevecz et al., 2004; Tu et al., 2005; Cho et al., 2017) and with cell-cycle progression (Cho et al., 1998; Spellman et al., 1998; Klevecz et al., 2004; Tu et al., 2005). These results are consistent with a model in which genome-wide transcriptional oscillation is independent of the CDK oscillator and coupled with other oscillators. Later genome-wide analysis, however, reported opposing conclusions (Orlando et al., 2008; Simmons Kovacs et al., 2008, 2012; Banyai et al., 2016; Rahi et al., 2016; Cho et al., 2017). The latest model proposes a more moderate “network” model, in which the transcriptional network is integrated in the CDK oscillator, at least in wild-type yeast cells (Figure 5D). Transcriptional factors regulate CDK activity primarily *via* controlling cyclin expression, whereas CDK activity regulates the activity of transcriptional factors in somatic cells (Cho et al., 2017). Thus, at least three molecular oscillators are coupled with CDK oscillator and are involved in CDC control (Figure 5D).

#### Molecular Mechanisms for Coupling Between Metabolic and Transcriptional Oscillators in Budding Yeast

Global transcriptional oscillation in budding yeast may interact in a feedback control mechanism with metabolic oscillation. Anabolic and catabolic metabolic enzymes are periodically synthesized under the control of a transcriptional oscillator. Global chromatin structure is regulated by the chromatin remodeling complex, whose activities are regulated *via* changes in concentrations of metabolites, such as acetyl-CoA and ATP (Cai et al., 2011; Machne and Murray, 2012; Amariei et al., 2013, 2014; Kuang et al., 2014; Mellor, 2016). An intriguing minimal model explains coordinated oscillation between transcription and metabolic oscillators by a double negative feedback loop mediated by two ATP-dependent nucleosome remodeling complexes (Machne and Murray, 2012; Amariei et al., 2013). Under conditions of high ATP availability, the RSC complex promotes transcription of genes encoding anabolic enzymes that consume ATP. By contrast, under conditions of low ATP availability, the Isw2 complex eventually promotes transcription of genes encoding catabolic enzyme genes that synthesize ATP. Therefore, global transcriptional oscillation and metabolic oscillation in budding yeast are interdependent (Figure 5D).

#### Molecular Mechanisms for Coupling Between Metabolic Oscillators and Circadian Transcriptional Oscillators in Mammalian Cells

The interdependent structure of transcriptional and metabolic oscillators in budding yeast appears to be conserved in the mammalian circadian oscillation of gene expression and metabolites (Figure 5D; Bellet and Sassone-Corsi, 2010; Reddy and Rey, 2014; Milev and Reddy, 2015; Mellor, 2016). Experiments have shown that negative feedback control in transcriptional regulation is sufficient for circadian transcriptional oscillation in mammals. However, ample evidence underlines the important contributions of metabolic oscillations for circadian transcriptional control in physiological situations. The expression of circadian genes is mediated by metabolites, such as NAD, acetyl CoA, and ATP, *via* chromatin remodeling complexes (Imai, 2010; Koike et al., 2012; Reddy and Rey, 2014; Milev and Reddy, 2015; Mellor, 2016; Milev et al., 2018). These studies suggest that the transcriptional oscillator in mammalian cells and budding yeast is coupled with the metabolic oscillator *via* chromatin remodeling complexes (Figure 5D).

#### Evolutionary Origin of Molecular Oscillators for Cell-Division Cycle Control

Yeast metabolic oscillation has regulatory properties that are similar to those of mammalian circadian oscillation, although their time scales of oscillation are different (Causton et al., 2015; Mellor, 2016). In budding yeast, metabolic oscillation shows circadian entrainment to temperature change with a 24-h period (Eelderink-Chen et al., 2010). Yeast metabolic oscillation maintains a certain rhythm even when temperature is changed (i.e., exhibits temperature compensation for reaction rate kinetics) (Kippert and Lloyd, 1995). In addition, yeast metabolic oscillation is regulated by circadian oscillation regulators, casein kinase 1 and glycogen synthase kinase 3 (Causton et al., 2015). Thus, budding yeast metabolic and mammalian circadian oscillators share regulatory molecules and system-level properties, prompting us to discuss the evolutionary origins of molecular oscillators for CDC control.

Phylogenetic analyses of the protein sequence of the budding yeast Cdk1 kinase Cdc28 indicated that CDKs must have evolved prior to the emergence of eukaryotes (Krylov et al., 2003). Moreover, knockout mice of core cell-cycle regulators, including cyclin Ds, Cdk4, Cdk6, cyclin E1, cyclin E2, and even Cdk2, are viable (reviewed in Sherr and Roberts, 2004). The CDK-cyclin system seems to have appeared in CDC evolution after the emergence of eukaryotes for sophistication and complexity of the system (Krylov et al., 2003); the masterful role of the CDK oscillator in CDC control becomes controversial.

The common evolutionary origin for biological time-keeping may be a redox oscillator (Murray et al., 2011; Reddy and Rey, 2014; Mellor, 2016; Milev et al., 2018). In addition to the redox states of NADH and glutathione, which are components of the metabolic oscillator, a wide range of redox systems is conserved in a broad range of living systems from bacteria to human (Reddy and Rey, 2014). Redox systems contribute to the system whereby photosynthetic bacteria release oxygen, and aerobic bacteria use oxygen for ATP synthesis. These bacteria, which first appeared 2–4 billion years ago, evolved defense systems to deal with the oxidizing cellular environment. Thus, the redox oscillator may be an ancient system and the core of the clustered oscillator for CDC. The coupling of the redox state for energy synthesis to the CDC (e.g., for temporal partitioning of oxidative stress) and the availability of cellular chemical components in coordination with CDC progression are consistent with the classic model, wherein the CDC is regulated by available cellular energy and synthesized components for cell duplication (Pederson, 2003). Thus, the CDC is under the control of the physiological state of the cell, which is determined based on energy synthesis and redox state, and the circadian clock. Taken together, the findings show that the CDC system is achieved by a concert of cellular physiology with the extracellular environment.

In summary, coupling between the CDK and transcriptional oscillators is achieved by periodic synthesis of CDC regulators from the transcriptional oscillator and feedback regulation of transcriptional control by the CDK oscillator (Figure 5D). Coupling between the metabolic and transcriptional oscillators is achieved by periodic production of metabolic regulators by the transcriptional oscillator and feedback regulation of the transcriptional oscillator by chromatin remodeling *via* metabolites (Figure 5D). Yeast transcriptional and mammalian circadian oscillators may play similar regulatory roles in coupling with the metabolic oscillator (Figure 5D).

The notion that the CDK oscillator serves as the central driver for CDC control is controversial. The CDK oscillator may instead “coordinate” cell-cycle progression and successful cell division by utilizing information, received from the other oscillators, about the cell’s physiological state, transcriptional state, and internal/external environments. On the other hand, as discussed in above, Kurz proposed a model in which the biochemical dynamics of the metabolic oscillator are controlled by mitochondrial geometry (Kurz et al., 2010, 2015). This model is similar to the allometric titration model, which proposed an interaction between oscillator dynamics and cellular geometry (e.g., balance between sizes of the cell and nucleus). In other words, physiological biochemical dynamics that are embedded in different organelles contain information about the relative sizes of the organelles and cellular geometry. By coupling cellular oscillators embedded in different organelles, biochemical reactions may proceed in concert with the relative cell and organelle sizes for successful cell division. Taken together, the models suggest that a cluster of molecular oscillators embedded in different cellular compartments may coordinate cellular physiology and geometry for successful cell proliferation or multicellular organization.

## Future Quantitative Studies for Cell-Division Cycle Control

Knowledge about the physiological significance of the clustered oscillator for CDC control may be expanded by experimental studies using various model organisms. We suggest two experiments using *Xenopus* embryos and one experiment using mammalian cells for further understanding of CDC control by a clustered oscillator embedded in different cellular compartments.

To study coupling between the metabolic and CDK oscillators, *Xenopus* embryos around MBT may be an interesting model system. Cell-cycle duration elongates in a power-law relation to cell size in mass resolution of single cells (Figure 4A). By contrast, in cells at the same developmental stage, cell-cycle durations were distributed in a multimodal peak with a 30-min period and exhibited quantization (Figure 3D; Masui and Wang, 1998; Wang et al., 2000). Thus, in *Xenopus* embryos after MBT, the power-law relation between cell-cycle duration and cell size coexists with quantized CDC control. To explain this quantized CDC control, Wang and Masui assumed a temporal unit production of MPF. The amount of MPF produced in a single 30-min round was sufficient for cell division before MBT, whereas the amount produced in multiple 30-min rounds was necessary for cell division after MBT, depending on the amount of the reduction in cell size. Involvement of the metabolic oscillator, or a candidate molecular system for quantized gating of CDC in mammalian cells (as discussed above), in a temporal unit production of MPF after MBT was not discussed (Klevecz, 1976; Papagiannakis et al., 2017). Metabolic oscillator dynamics in mitochondria are thought to depend on mitochondrial geometry (Kurz et al., 2015). One interesting topic for future research is how the temporal dynamics of the mitochondrial metabolic oscillator changes according to changes in the fractal structure of mitochondria in *Xenopus* embryos around MBT.

To study coupling between the circadian transcriptional and CDK oscillators, *Xenopus* embryos after MBT may be an interesting model system. At MBT, when somatic transcription initiates (Newport and Kirschner, 1982b), the transcriptional oscillator potentially starts to be integrated into the CDK oscillator, as described by the integration model (Figure 5D; Cho et al., 2017). Theoretical studies have shown that variability and temporal correlation of cell-cycle duration are due to coupling between oscillators showing large differences in the periods of the cycle (Lloyd et al., 1992). These chaotic properties of cell-cycle duration may be generated in accordance with maturation of the circadian transcriptional oscillator during animal development. Expression of circadian genes in an embryonic organ or tissue precedes the initiation of the 24-h periodic expression of those genes in mice (Dolatshad et al., 2010), rats (Sladek et al., 2004, 2007), zebrafish (Delaunay et al., 2000), and *Xenopus* embryos (Green et al., 2001). Oscillations of circadian gene expressions start as an ultradian rhythm during early embryonic cell divisions in zebrafish (Delaunay et al., 2000) and during somitogenesis in *Xenopus* embryos (Curran et al., 2008, 2014). One interesting topic of study would be how the maturation of circadian transcriptional oscillator affects the generation of chaotic CDC control in a relation to cell-size changes during development.

Mammalian somatic cells might be useful for studying applicability of the allometric titration model. As discussed above, there were two different mechanisms of CDC control based on changes in cell size. In budding and fission yeasts, cells arrested CDC progression at the G1 or G2 phase until cell size reached a certain volume; this process is classified as the arrest-or-go mechanism for cell-size maintenance. By contrast, cell size-dependent CDC control utilizes length control of cell-cycle phases in animal embryos (Iwao et al., 2005; Arata et al., 2014), similar to mammalian cells (Tzur et al., 2009). It would be interesting to apply the allometric titration model to understand the mechanism of cell-size control in mammalian cells. Applicability of the allometric titration model to mammalian cells can be tested in experiments where the ratio between cell size and nuclear size is changed at the power of at least two. However, because it is difficult to manipulate either cell size or nuclear size in a broad range in the power of at least two, the combination of experimental manipulations to reduce and increase cell size and nuclear size may be effective when performed in fission yeast (Neumann and Nurse, 2007).

## Conclusions

Molecular biological studies of CDC control have shown that the CDK oscillator is the core for CDC control, which explains the oscillatory property of CDC, but this oscillator is not sufficient for explaining statistical distributions of cell-cycle duration. Based on experimental research about molecular oscillators with faster or slower rhythms than the CDC, this review identifies a model showing that the cell cycle is controlled by a chaotic dynamical system due to a cluster of coupled oscillators. This model consistently explains the statistical distributions accounting for the variability of cell-cycle duration. Additionally, the chaotic dynamics inherent in the metabolic oscillator (Roussel and Lloyd, 2007) and thereby the chaotic dynamics generated by the coupled oscillator system are multilayered for CDC control. On the other hand, based on experimental research on the relationship between different dimensions, such as cell-cycle duration and spatial size or structures, we derived a model showing that biochemical reactions for CDC control are controlled by the spatial size and structures of cells and mitochondria. Wang and Masui, Arata et al., and Kurz et al. showed the quantitative relationships between biochemical dynamics and spatial size or structures (Masui and Wang, 1998; Wang et al., 2000; Arata et al., 2014; Kurz et al., 2015). These studies raised the possibility that the relationship of circadian dynamics and transcription dynamics with the spatial size of cells and mitochondria may also have some certain constraints. Among these quantitative relationships of temporal dynamics and spatial structure of cells, it may be possible to discover a “state equation of CDC control,” as if the ideal gas law is derived from Boyle’s and Charles’ laws. This state equation would consistently constrain the spatiotemporal relationships for CDC control and would describe and predict the temporal dynamics of the CDC in various cell types of different organisms. Thus, CDC dynamics can be described by dynamical system equation and the state equation of CDC control.

Dynamical system equation for CDC would define physiological states of normal or cancer cells *via* stability, bifurcation and attractor analyses, which serve as a theoretical basis for predicting cell proliferation and artificial control of the CDC for clinical treatments. On the other hand, state equation of CDC control may represent a general restriction of cells or design principle of cells that governs cells for proliferation. This design principle of cell proliferation may provide a guideline for artificial synthesis of living cell. Additionally, scale-free temporal dynamics could theoretically be generated in some chaotic dynamical system equations under certain conditions (Ott, 2002). Therefore, it is possible that scale-free dynamics in CDC control may be derived from a mixture of a fractal structures in cellular space and the biochemical chaotic dynamical systems (Figures 5D,E). Dynamical system equation for CDC control and state equation of CDC control would have the theoretical consistency, as if Kepler’s law is derived from Newton’s equation of motion with gravitational force. The relationship between the two equations may even ultimately contribute to revealing a critical mechanism for stable self-replication and self-organization of cells and organisms.

## Author Contributions

YA contributed to this manuscript in terms of conception of the work and drafting the work. HT contributed to terms of conception of the work and revising it critically for important intellectual content, and analyses in Figures 4E,F, 5A,B.

### Conflict of Interest Statement

The authors declare that the research was conducted in the absence of any commercial or financial relationships that could be construed as a potential conflict of interest.
